# Artificial Butter

**Published:** 1888-11

**Authors:** 


### ARTIFICIAL BUTTER.

Gustavus F. Swift, of the firm of Swift & Co., of the town of Lake, (near Chicago), describes as follows the method in use in the manufacture of artificial butter by his company :

The fat is taken from the cattle in the process of slaughtering, and after thorough washing is placed in a bath of clean, cold water and surrounded with ice, where it is allowed to remain until all animal heat has been removed. It is then cut into small pieces by machinery and cooked at a temperature of about 150 degrees, until the fat in liquid form has separated from the fibrane or tissue ; then settled until it is perfectly clear. Then it is drawn into draining vats and allowed to stand a day, when it is ready for the presses. The pressing extracts the stearine, leaving the remaining product, which is commercially known as oleo oil, which, when churned with cream or milk or both, and with usually a portion of creamery butter, the whole being properly salted, gives the the new food product, oleomargarine.

In making butterine, we use neutral lard, which is made from selected leaf-lard in a very similar manner to oleo oil, excepting that no stearine, is extracted. This neutral lard is cured in salt brine for forty-eight to seventy hours at an ice-water temperature. It is then taken, and with the desired proportion of oleo oil and fine butter, is churned with cream and milk. producing an article, which, when properly salted and packed, is ready for market.

In both cases coloring matter is used which is the same as that used by dairymen to color their butter. At certain seasons of the year, viz, in cold weather, a small quantity of sesame oil, or salad oil made from cotton seed, is used to soften the texture of the product.

On the subjest of the wholesomeness of artificial butter, there is a large difference of opinion. It is undoubtedly true that a great deal of artificial butter has been thrown upon the market that has been carelessly made, and therefore harmful to the health. On the other hand, that a butter substitute, made carefully out of the fat of a perfectly healthy bullock or swine, is not prejudicial to health, has the endorsement of some of our eminent chemists who have given the subject careful attention.

